# Morning chronotype and digestive tract cancers: Mendelian randomization study

**DOI:** 10.1002/ijc.34284

**Published:** 2022-09-12

**Authors:** Shuai Yuan, Amy M. Mason, Olga E. Titova, Mathew Vithayathil, Siddhartha Kar, Jie Chen, Xue Li, Stephen Burgess, Susanna C. Larsson

**Affiliations:** 1 Unit of Medical Epidemiology, Department of Surgical Sciences, Uppsala University, Uppsala, Sweden; 2 Unit of Cardiovascular and Nutritional Epidemiology, Institute of Environmental Medicine, Karolinska Institutet, Stockholm, Sweden; 3 British Heart Foundation Cardiovascular Epidemiology Unit, Department of Public Health and Primary Care, University of Cambridge, Cambridge, UK; 4 MRC Cancer Unit, University of Cambridge, Cambridge, UK; 5 MRC Integrative Epidemiology Unit, Bristol Medical School, University of Bristol, Bristol, UK; 6 Centre for Global Health, Zhejiang University School of Medicine, Hangzhou, China; 7 Department of Big Data in Health Science School of Public Health, Center of Clinical Big Data and Analytics of The Second Affiliated Hospital, Zhejiang University School of Medicine, Hangzhou, China; 8 MRC Biostatistics Unit, University of Cambridge, Cambridge, UK; 9 Department of Public Health and Primary Care, University of Cambridge, Cambridge, UK

**Keywords:** chronotype, colorectal cancer, digestive system cancer, gastric cancer, Mendelian randomization

## Abstract

Morning chronotype has been associated with a reduced risk of prostate and breast cancer. However, few studies have examined whether chronotype is associated with digestive tract cancer risk. We conducted a Mendelian randomization (MR) study to assess the associations of chronotype with major digestive tract cancers. A total of 317 independent genetic variants associated with chronotype at the genome-wide significance level (*P* < 5 × 10 ^–8^) were used as instrumental variables from a genome-wide meta-analysis of 449 734 individuals. Summary-level data on overall and six digestive tract cancers, including esophageal, stomach, liver, biliary tract, pancreatic and colorectal cancers, were obtained from the UK Biobank (11 952 cases) and FinnGen (7638 cases) study. Genetic liability to morning chronotype was associated with reduced risk of overall digestive tract cancer and cancers of stomach, biliary tract and colorectum in UK Biobank. The associations for the overall digestive tract, stomach and colorectal cancers were directionally replicated in FinnGen. In the meta-analysis of the two sources, genetic liability to morning chronotype was associated with a decreased risk of overall digestive tract cancer (odds ratio [OR] 0.94, 95% confidence interval [CI]: 0.90-0.98), stomach cancer (OR 0.84, 95% CI: 0.73-0.97) and colorectal cancer (OR 0.92, 95% CI: 0.87-0.98), but not with the other studied cancers. The associations were consistent in multivariable MR analysis with adjustment for genetically predicted sleep duration, short sleep, insomnia and body mass index. The study provided MR evidence of inverse associations of morning chronotype with digestive tract cancer, particularly stomach and colorectal cancers.

## 1 Introduction

Digestive tract cancer with 4.8 million new cases (26% of global cancer incidence) caused 3.4 million premature deaths (35% of cancer-related mortality) in 2018 worldwide, which poses a large global disease burden.^1^ Previous studies identified several risk factors for digestive tract cancer, including obesity, cigarette smoking, alcohol consumption and hepatitis B virus infection.^1–5^ Primary and secondary prevention strategies targeting at above risk factors may play an important role in preventing and controlling gastrointestinal malignancies.^1^ However, except for the above traditional risk factors, novel factors are scarcely studied, such as chronotype, possibly influencing the risk of gastrointestinal cancers.

Chronotype is the natural propensity for the individual to sleep at a particular time. Two extreme types of chronotype, termed morningness (an early bird) and eveningness (a night owl), means having an advanced and delayed sleep period, respectively. Evening chronotype associates with less physical activity^6^ and unhealthy dietary habits^7^ and thus could potentially have adverse health effects and increase the risk of cancer.^8^ A matched case-control study revealed that patients with gastroenteropancreatic neuroendocrine tumors had more commonly an evening chronotype compared to healthy controls.^9^ Another study using metagenomic sequencing analysis found that morning chronotype was associated with a decreased abundance of *Alistipes* and an elevated abundance of *Lachnospira*.^10^ These two gut microbiome genera may play a role in the development of cancers in the gastrointestinal tract, such as colorectal cancer.^11,12^ Even though a few studies linked chronotype to digestive tract cancer, no studies examined this association.

Mendelian randomization (MR) is an epidemiological approach that can strengthen the causal inference by utilizing genetic variants as instrumental variables for an exposure.^13^ The design has two merits. First, it can minimize confounding effects since genetic variants are randomly assorted at conception and therefore not correlated with environmental or self-adopted factors that are usually confounders in the association between the exposure and outcome.^13^ Second, the approach can diminish reverse causality because the onset and progression of disease cannot modify the germline genotype.^13^ Previous MR studies revealed inverse associations of morningness with the risk of prostate and breast cancers.^14–16^ Nevertheless, no studies were conducted to examine the associations between chronotype and digestive tract cancers. Here, we conducted an MR investigation to explore the causality of these associations.

## 2 Methods

### 2.1 Outcome data sources

Genetic associations with six digestive tract cancers (esophageal, stomach, liver, biliary tract, pancreatic and colorectal cancers) and overall digestive tract cancer were obtained from the UK Biobank study and the FinnGen study.^17^ The UK Biobank study is an ongoing cohort study that was initiated by recruiting about 500 000 adults between 2006 and 2010. Our study was based on data from a total of up to 367 542 individuals followed up until April 2022 after removal of those with non-Caucasian ethnicity (to reduce population stratification bias), sex mismatch, excess heterozygosity and low genotype call rate, and those related by third degree or higher. We defined 11 952 digestive tract cancer cases including 1339 esophageal, 1086 stomach, 503 liver, 656 biliary tract, 1414 pancreatic and 7543 colorectal cancer patients. These cases were diagnosed by using codes of International Classification of Diseases (9th and 10th revisions) and self-reported information verified by interview with a nurse from national registries (Table S1). For the FinnGen study, we used data from the R6 release that includes 309 154 Finnish individuals after excluding participants with ambiguous gender, high genotype missingness (>5%), excess heterozygosity (±4 SD) and non-Finnish ancestry.^17^ A total of 7638 patients with digestive tract cancers were ascertained, including 358 esophageal, 889 stomach, 442 liver, 157 biliary tract, 881 pancreatic and 4401 colorectal cancer patients. Similarly, cancer cases were defined by codes of International Classification of Diseases (8th, 9th and 10th revisions) with information from nationwide registries (Table S2). The associations were adjusted for age, sex and 10 genetic principal components in both data sources.

### 2.2 Genetic instrument selection

Single-nucleotide polymorphisms (SNPs) associated with chronotype at the genome-wide significance level (*P* < 5 × 10 ^−8^) were extracted from a genome-wide meta-analysis of 449 734 individuals of European-ancestry from the UK Biobank and 248 098 individuals of European-ancestry from 23andMe.^18^ Linkage disequilibrium was estimated among selected SNPs based on the 1000 Genomes European reference panel.^19^ SNPs in linkage disequilibrium (*r*^2^ > .01) were excluded and the SNP with the smallest *P* value for the genetic association with chronotype was retained. After clumping, 317 SNPs in autosomal chromosomes were selected as instrumental variables (Table S3). The genetic score of these SNPs showed no associations with sleep duration and quality as well as insomnia.^18^ In the main analysis, we used the estimates (beta and corresponding SE coefficients) of the associations between SNPs and chronotype from the meta-analysis of UK Biobank and 23andMe to increase the power. In addition, we used the genetic association estimates obtained from only 23andMe to minimize the sample overlap in the sensitivity analysis (Table S3). Genetic associations were scaled to one point increase in chronotype category (–2 for definitely evening, –1 for more evening than morning, 0 for unknown, 1 for more morning than evening and 2 for definitely morning) and estimated by a linear mixed model with adjustment for age, sex, study center, genotyping array and genetic principal components.^18^

### 2.3 Sleep features and body mass index data sources

Summary-level data on sleep duration and short sleep (<7 hours/day) were obtained from a genome-wide association analysis of 446 118 individuals of European ancestry (106 192 short sleep cases compared to 305 742 controls with 7- to 8-hour sleep duration).^20^ Genetic associations were adjusted for age, sex, 10 principal components of ancestry, genotyping array and genetic correlation matrix.^20^ Summary-level data on insomnia were extracted from a genome-wide association study of 453 379 individuals of European ancestry (345 022 cases with any insomnia symptoms and 108 357 controls).^21^ Genetic associations were adjusted for age, sex, 10 principal components of ancestry and genotyping array.^21^ Genetic associations with body mass index (BMI) were extracted from the genome-wide analysis in the Genetic Investigation of ANthropometric Traits consortium including 806 834 individuals with adjustment for age, sex and genetic principal components.^22^

### 2.4 Statistical analysis

We used the random-effects multiplicative inverse variance weighted method as the primary analysis to estimate the association between genetic liability to chronotype and the risk of digestive tract cancers. Given that the analysis is sensitive to outliers and horizontal pleiotropy, four sensitivity analyses, including the weighted median,^23^ MR-Egger,^24^ MR-PRESSO^25^ and contamination mixture^26^ methods, were utilized to examine the consistency of the results and detect horizontal pleiotropy if any. We described assumptions and strengths of above methods in Table 1. Heterogeneity across estimates of SNPs for one association was assessed by Cochran’s *Q* value. The MR-Egger intercept test was used to examine horizontal pleiotropy (*P* < .05). Possible outliers were identified by MR-PRESSO analysis. Given that sleep features, short sleep in particular, were associated with the risk of several digestive tract cancers,^27^ we performed multivariable MR analysis with adjustment for genetically predicted sleep duration and genetic liability to having short sleep and insomnia to reduce the corresponding pleiotropy. Considering that high BMI is an important risk factor for digestive tract cancers,^5^ we also conducted multivariable MR analysis with adjustment for genetically predicted BMI to assess potential mediating effects of BMI on digestive tract cancer risk. We used the same set of instrumental variables (317 SNPs) as the univariable MR analysis in multivariable MR analysis. Beta and SE coefficients of the associations of SNPs with genetic liability to chronotype, sleep traits or BMI and cancers were regressed to obtained multivariable MR estimates using the multivariable random-effects multiplicative inverse variance weighted method. The Benjamini–Hochberg false discovery rate correction was used to account for multiple comparisons.^28^ All tests were two-sided and performed using the TwoSampleMR, MR-PRESSO and MendelianRandomization packages in the R software (version 4.0.2).

## 3 Results

**Figure 1:**
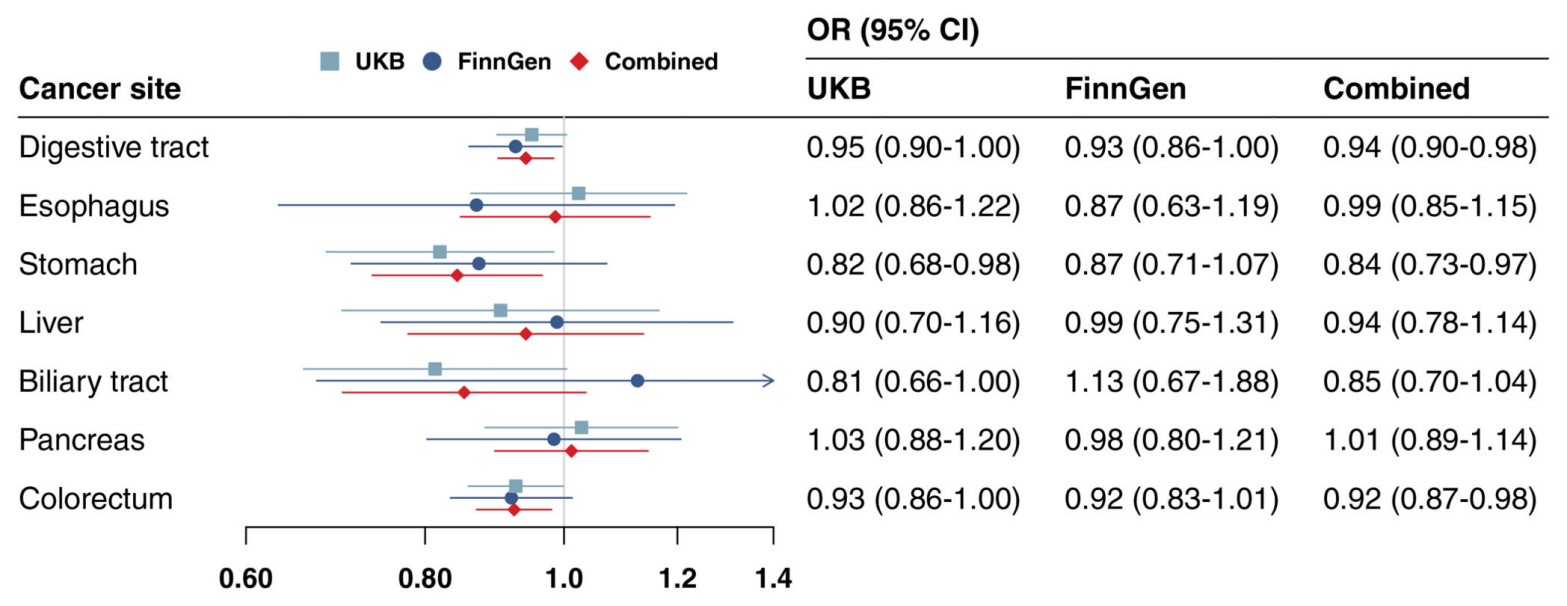
Associations of genetic liability to morning chronotype with risk of digestive tract cancers in the UK Biobank (UKB) and FinnGen study. CI, confidence interval; OR, odds ratio. The OR was scaled to genetically predicted one score increase in chronotype phenotype coded from –2 to 2, which indicates definitely evening to definitely morning [Color figure can be viewed at wileyonlinelibrary.com]

Genetic liability to morning chronotype was associated with a decreased risk of overall digestive tract cancer (odds ratio [OR] 0.95, 95% confidence interval [CI]: 0.90-1.00), stomach cancer (OR 0.82, 95% CI: 0.68-0.98), biliary tract cancer (OR 0.81, 95% CI: 0.66-1.00) and colorectal cancer (OR 0.93, 95% CI: 0.86-1.00) in the UK Biobank study (Figure 1). The associations for cancers of overall digestive tract, stomach and colorectum but not for biliary tract cancer were directionally consistent in the FinnGen study (Figure 1). In the combined analysis of the two data sources, genetic liability to morning chronotype was associated with a decreased risk of overall digestive tract cancer (OR 0.94, 95% CI: 0.90-0.98), stomach cancer (OR 0.84, 95% CI: 0.73-0.97) and colorectal cancer (OR 0.92, 95% CI: 0.87-0.98). All three associations persisted after correction for multiple testing (Table S4). Genetic liability to being a morning person was not associated with cancers of the esophagus, liver or pancreas (Figure 1).

The associations remained consistent but with wider CIs in the sensitivity analyses (Table 2). Mild-to-moderate heterogeneity across SNPs’ estimates was observed in all analyses. We observed no indication of horizontal pleiotropy in MR-Egger intercept test (Table 2, *P* > .05). One outlier was identified in MR-PRESSO analysis of colorectal cancer in UK Biobank and of biliary tract cancer in FinnGen; however, the associations were stable after removal of the outlier (Table 2). The associations remained stable in the analysis where estimate coefficients of SNP-chronotype associations were obtained from only the 23andMe population (Table S5). The associations for overall digestive tract cancer, stomach cancer and colorectal cancer also remained stable in multivariable MR analysis with adjustment for genetically predicted sleep duration and BMI and genetic liability to having short sleep and insomnia (Table 3).

## 4 Discussion

We conducted an MR study to examine the associations between genetically proxied chronotype and risk of overall and six site-specific digestive tract cancers in UK Biobank and FinnGen. We found robust inverse associations of genetic liability to morning chronotype with risk of overall digestive tract cancer, stomach cancer and colorectal cancer. The associations were consistent in both data sources and remained stable after adjustment for sleep duration, short sleep and insomnia. BMI appeared not to mediate the above associations. Of note, these findings relate to a naturally morning chronotype instead of a morning behavior (eg, an evening type may be forced to behave like a morning type due to social reasons such as work, despite sleeping hours).

As a novel health-related factor, chronotype has been studied in relation to cancer risk in a few studies in recent decades. Observational evidence is consistent on the association between morning chronotype and reduced risk of breast and prostate cancers.^29,30^ These associations were further strengthened in MR studies.^14–16,31^ Being an early bird was also associated with the lower risk of other hormone-related cancers, like ovarian and endometrial cancers,^32,33^ and lung cancer.^34^ However, little data were available on association between chronotype and digestive tract cancer. A case-control study including a total of 318 participants found that patients with gastroenteropancreatic neuroendocrine tumors had a higher percentage of having evening chronotype compared to the healthy controls.^9^ That study further showed that a higher chronotype score indicating a higher adherence to morningness was also associated with lower risk of metastasis, grading G2 and progressive disease.^9^ Even though the study controlled for several important confounders, like age, sex and BMI, the observed associations might still be biased by residual confounding, which hindered the examination on the causal impact of chronotype on gastroenterological cancer. Our MR results supported this observational study and strengthened the causality of the inverse association between morning chronotype and digestive tract cancer. Notably, we further examined the associations with gastrointestinal tract cancers by site and found strong effects on stomach and colorectal cancers. We also observed an inverse association between morning chronotype and biliary tract cancer in UK Biobank, but not in the combined analysis, which was caused by the heterogeneous association observed in FinnGen. Thus, larger studies are needed to further explore the association between morning chronotype and biliary tract cancer.

Some underlying mechanisms in support of morningness and the reduced risk of digestive tract cancer have been proposed albeit scarcely examined. First, chronotype has been linked to the profile of gut microbiota that may influence the development of digestive tract cancer.^10,35^ A detailed appraisal of mediation of gut microbiota in the association between chronotype and digestive tract cancer is of importance for clinical and therapeutic work. Second, genetic liability to morning chronotype has been associated with increased intake of foods known to constitute a healthy diet (eg, fresh fruit and bran cereals) and lower intake of unhealthy foods and beverages (eg, processed meat and beer).^36^ Those foods are associated with risk of digestive tract cancers, particularly stomach and colorectal cancer.^37,38^ Third, evening chronotype has been associated with DNA methylation of certain gene sites, like *BACH2, JRK* and *RPS6KA2*, which are related to the cancer development.^39^ Fourth, evening chronotype may be associated with impaired metabolic homeostasis, like insulin resistance and increased levels of low-density lipoprotein,^40^ which have been associated with an increased risk of colorectal cancer.^41,42^ In addition, increased levels of BMI have been proposed to be a possible mediator in the association between chronotype and digestive tract cancer since people with morning preference have a decreased frequency of obesity^43^ that is a causal risk factor for digestive tract cancers.^5^ However, we found no evidence that our findings were mediated by BMI.

Chronotype can be influenced by genetic and environmental factors including cultural and social influences, urban lifestyle, exposure to light, sleep schedule and so on.^44^ Given the protective effects of a morning chronotype on several cancer outcomes,^14–16,31^ strategies that formulate a morning type, like limiting evening screen time and maintaining a regular sleep schedule, should be promoted to reduce the disease burden of these cancers. In addition, chronotype may influence the disease risk via altering diet preference and pattern.^8^ For example, individuals with an evening type were more likely to skip breakfast, delay mealtime and increase calorie intake during night and alcohol consumption compared to those with a morning chronotype.^7^ Even though it is unclear that which diet-related pathway mediates the association between chronotype and digestive tract cancer, this finding implies that (a) chronotype and diet information should be considered when estimating risk of digestive cancer; and (b) a diet intervention may be an efficient strategy to lower risk of digestive tract cancer among individuals with an evening chronotype.

The study has several strengths. The major one is MR design that strengthened the causal inference by minimizing confounding. We combined data from two large-scaled biobanks, which increased the power of the analysis. In addition, the consistency of results from two sources indicated the robustness of our findings. We conducted multivariable MR analysis and ruled out the possibility that our MR results might be biased by pleiotropic effects of sleep-related features. The current analysis was confined to the European population, which reduced the population structure bias. Several limitations deserve consideration when interpreting our results. First, the population confinement to the European population may limit the generalizability of our findings to other populations. Second, we may overlook the moderate associations due to inadequate power caused by a small number of cases for certain cancers even though we combined two data sources. Third, the interplay of chronotype and diet cannot be studied in our study based on summary-level statistic data.^44^

In summary, this MR study found the inverse associations between morning chronotype and digestive tract cancers, in particular cancers of stomach and colorectum. These findings suggest a causal role of being an early bird instead of a night owl in lowering digestive tract cancer. The mechanism behind these associations needs further study.

## Supplementary Material

Supplementary File

## Figures and Tables

**Table 1:** Assumptions and strengths of used Mendelian randomization analyses

Method	Assumptions	Strengths
The inverse variance weighted method	No unbalanced horizontal pleiotropy	Provides the most precise estimate if all instruments are valid
The weighted median method	More than 50% of weight from valid genetic instruments	Informs about the estimate supported by the majority of evidence
MR-Egger regression	Associations of the genetic instruments with the exposure are uncorrelated with any pleiotropic effects of the instruments on the outcome	Provides consistent estimate under this assumption
MR-PRESSO	The largest group of candidate instruments with similar estimates is the group of valid instrumental variables.	Detects outliers and provides estimate after removal of outliers
Contamination mixture method	Plurality of instruments are valid	Informs about the estimate supported by the plurality of evidence (ie, the estimate with the greatest amount of support)

**Table 2:** Associations of genetic liability to morning chronotype with digestive tract cancers in MR sensitivity analyses

					Weighted median			MR-Egger			Contamination mixture			MR-PRESSO		
Cancer	Cases	Controls	Cochran’s *Q*	*P*_intercept_	OR	95% CI	*P*	OR	95% CI	*P*	OR	95% CI	*P*	OR	95% CI	*P*
*UK Biobank*																
Digestive tract	11 952	270 458	320	.963	0.93	0.86-1.01	.099	0.95	0.81-1.11	.547	0.94	0.88-1.00	.078	0.95	0.90-1.00	.069
Esophagus	1339	366 203	368	.835	1.11	0.85-1.46	.447	0.98	0.60-1.59	.921	1.05	0.78-1.38	.662	1.02	0.86-1.22	.789
Stomach	1086	366 456	332	.530	0.82	0.62-1.08	.162	0.70	0.42-1.17	.179	0.66	0.49-0.90	.010	0.82	0.68-0.98	.033
Liver	503	367 039	300	.710	0.80	0.53-1.21	.297	0.79	0.38-1.65	.535	0.61	0.20-1.49	.312	0.90	0.70-1.16	.433
Biliary tract	656	366 886	269	.821	0.73	0.51-1.04	.082	0.76	0.40-1.44	.400	0.77	0.49-1.07	.129	0.81	0.66-1.00	.056
Pancreas	1414	366 128	310	.937	1.09	0.84-1.41	.528	1.05	0.67-1.62	.842	1.23	0.99-1.51	.068	1.03	0.88-1.20	.722
Colorectum	7543	359 999	393	.697	0.91	0.81-1.01	.077	0.96	0.78-1.19	.733	0.84	0.78-0.92	.001	0.92	0.85-0.99	.030
*FinnGen*																
Digestive tract	7638	204 070	301	.675	0.91	0.81-1.02	.103	0.89	0.72-1.09	.258	0.92	0.84-1.01	.091	0.93	0.86-1.00	.041
Esophagus	358	204 070	294	.440	0.68	0.40-1.13	.139	0.63	0.26-1.52	.302	0.34	0.21-0.76	.014	0.87	0.63-1.19	.387
Stomach	889	204 070	297	.545	0.96	0.70-1.33	.813	1.03	0.58-1.81	.927	1.07	0.71-1.58	.789	0.87	0.71-1.07	.193
Liver	442	204 070	280	.669	0.91	0.58-1.43	.687	0.84	0.38-1.87	.670	0.35	0.22-1.43	.076	0.99	0.75-1.31	.937
Biliary tract	157	204 070	340	.131	1.37	0.64-2.93	.423	3.13	0.76-12.9	.116	0.00	0.00-41.3	NA	1.16	0.70-1.94	.557
Pancreas	881	204 070	289	.073	0.78	0.57-1.06	.117	0.60	0.34-1.07	.085	0.74	0.54-1.02	.068	0.98	0.80-1.21	.877
Colorectum	4401	204 070	305	.763	0.94	0.81-1.08	.372	0.96	0.73-1.25	.738	0.84	0.76-0.94	.004	0.92	0.83-1.01	.090

Abbreviations: CI, confidence interval; OR, odds ratio. *Note:* The *P*_intercept_ is the *P* for MR-Egger intercept test and a *P*_intercept_ < .05 indicates horizontal pleiotropy. One outlier was identified in MR-PRESSO analysis of colorectal cancer in UK Biobank and that of biliary tract cancer in FinnGen.

**Table 3:** Results of multivariable MR analysis with adjustment for sleep duration, short sleep, insomnia and BMI in the meta-analysis of UK Biobank and FinnGen

Cancer	Model	OR	95% CI	*P* value
Digestive tract cancer	Univariable MR	0.94	0.90-0.98	.007
	MVMR adjusted for sleep duration	0.94	0.90-0.98	.009
	MVMR adjusted for short sleep	0.94	0.90-0.99	.010
	MVMR adjusted for insomnia	0.94	0.90-0.98	.008
	MVMR adjusted for BMI	0.94	0.90-0.98	.008
Stomach cancer	Univariable MR	0.84	0.73-0.97	.014
	MVMR adjusted for sleep duration	0.84	0.74-0.97	.015
	MVMR adjusted for short sleep	0.84	0.73-0.97	.015
	MVMR adjusted for insomnia	0.84	0.73-0.96	.013
	MVMR adjusted for BMI	0.84	0.73-0.96	.013
Colorectal cancer	Univariable MR	0.92	0.87-0.98	.009
	MVMR adjusted for sleep duration	0.93	0.87-0.98	.013
	MVMR adjusted for short sleep	0.93	0.87-0.98	.013
	MVMR adjusted for insomnia	0.92	0.87-0.98	.012
	MVMR adjusted for BMI	0.93	0.87-0.98	.012

Abbreviations: BMI, body mass index; CI, confidence interval; MR, Mendelian randomization; MVMR, multivariable Mendelian randomization; OR, odds ratio.

## Data Availability

This work has been conducted using the UK Biobank Resource. The UK Biobank is an open access resource and bona fide researchers can apply to use the UK Biobank dataset by registering and applying at http://ukbiobank.ac.uk/register-apply/. Further information is available from the corresponding author upon request.

## What’s new?

Cancers of the digestive tract are associated with various traditional risk factors, including smoking and obesity. A possible novel risk factor may be chronotype, an individual’s natural tendency to sleep at a particular time. In our study, the authors assessed potential associations between digestive tract cancers and chronotype using Mendelian randomization analysis of UK Biobank and FinnGen study data. Overall risk of digestive tract cancer, stomach cancer and colorectal cancer was found to be inversely associated with genetic liability to morning chronotype. Analyses further indicate that the associations are independent of sleep duration, short sleep, insomnia and body mass index.

## Abbreviations

BMI: body mass index
CI: confidence interval
MR: Mendelian randomization
OR: odds ratio
SNP: single-nucleotide polymorphisms
