# Supplementary material for: Morning chronotype and digestive tract cancers: Mendelian randomization study
Source: Int J Cancer. 2022 Sep 22;152(4):697–704. doi: 10.1002/ijc.34284 (PMC7613990; doi:10.1002/ijc.34284)
Supplement: Supplementary file 1 — Appendix S1 Supporting Information. [file IJC-152-697-s001.pdf]

Supporting information for

Morning chronotype and digestive tract cancers: Mendelian randomization study

Shuai Yuan, Amy M. Mason, Olga E. Titova, Mathew Vithayathil, Siddhartha Kar, Jie Chen, Xue Li, Stephen Burgess, Susanna C. Larsson

Table of Contents

Supplementary Table 1. Diagnostic information of cancer in UK Biobank **2**

Supplementary Table 2. Diagnostic information of cancer in FinnGen **3**

Supplementary Table 3. Genetic instrumental variables used for genetic liability to chronotype **4**

Supplementary Table 4. Results of multiple testing correction for combined associations by the false discovery rate (FDR) method **13**

Supplementary Table 5. Associations between genetic liability to chronotype and cancers of digestive tract, stomach, and colorectum in the analysis where the exposure data were obtained from 23andMe **14**

Supplementary Table 1. Diagnostic information of cancer in UK Biobank

Cancer site/cancer	Diagnostic information		
	**ICD-9 codes**	**ICD-10 codes**	**Self-reported cancer**
Digestive system cancer	150, V10.03, 151, V10.04, 157, 155, 156, 153, 154.0, 154.1, V10.05, V10.06	C15, Z85.01, C16, Z85.028, C25, Z85.07, C22.0, C22.1, C23.X, C24.X, C18, C19, C20, Z85.038, Z85.048	1017, 1018, 1034, 1024, 1025, 1020, 1022, 1023
Esophagus	150, V10.03	C15, Z85.01	1017
Stomach	151, V10.04	C16, Z85.028	1018
Liver	155	C22.0	1024
Biliary tract	155.1, 156.0	C22.1, C23.X, C24.X	1025
Pancreas	157	C25, Z85.07	1034
Colorectum	153, 154.0, 154.1, V10.05, V10.06	C18, C19, C20, Z85.038, Z85.048	1020, 1022, 1023

ICD, International Classification of Diseases.

Supplementary Table 2. Diagnostic information of cancer in FinnGen

Cancer site/cancer	Diagnostic information		
	ICD-8 codes	ICD-9 codes	ICD-10 codes
Digestive system cancer	150, 151, 152, 153, 1540, 1541, 1542, 155, 1560, 156, 157, 158, 159	150, 151, 152, 153, 154, 155, 156, 157, 158, 159	C15, C16, C17, C18, C19, C20, C21, C22, C23, C24, C25, C26
Esophagus	150	150	C15
Stomach	151	151	C16
Liver	155	155	C22
Biliary tract	156	156	C24
Pancreas	157	157	C25
Colorectum	153, 1540, 1541	153, 154	C18, C19, C20

ICD, International Classification of Diseases.

Supplementary Table 3. Genetic instrumental variables used for genetic liability to chronotype

SNP	Chr	Position	EA	EAF	Beta_meta	SE_meta	Beta_23	SE_23
rs10916892	1	21201325	T	0.62	-0.035	0.004	-0.036	0.006
rs11102807	1	115061584	A	0.54	-0.022	0.004	-0.019	0.006
rs11165655	1	96959104	A	0.53	-0.028	0.004	-0.029	0.006
rs11208844	1	66851147	A	0.14	-0.029	0.005	-0.032	0.008
rs1144566	1	182569626	T	0.03	0.231	0.010	0.312	0.018
rs115073088	1	174215858	A	0.98	-0.076	0.011	-0.077	0.018
rs11588913	1	79963816	A	0.40	-0.024	0.004	-0.021	0.006
rs12040629	1	77705365	A	0.16	0.073	0.005	0.083	0.008
rs12065331	1	14507831	T	0.31	-0.024	0.004	-0.030	0.007
rs12140153	1	62579891	T	0.09	-0.060	0.007	-0.068	0.012
rs1221502	1	193276975	A	0.74	0.020	0.003	0.023	0.007
rs17448682	1	15966713	T	0.23	0.035	0.004	0.035	0.007
rs17575798	1	110086451	A	0.19	-0.034	0.004	-0.034	0.008
rs4657983	1	195454557	A	0.65	-0.025	0.004	-0.031	0.006
rs481214	1	93469865	A	0.61	0.023	0.004	0.020	0.006
rs5016898	1	81672013	T	0.42	-0.024	0.004	-0.029	0.006
rs61773390	1	7884525	T	0.19	0.066	0.005	0.077	0.008
rs6429233	1	241137033	A	0.46	0.020	0.003	0.022	0.006
rs6665637	1	153756083	A	0.28	-0.020	0.003	-0.027	0.007
rs6690292	1	113188419	T	0.73	-0.025	0.004	-0.025	0.007
rs72720396	1	91191582	A	0.77	-0.042	0.004	-0.037	0.007
rs9436119	1	150467753	A	0.38	0.040	0.003	0.053	0.006
rs975025	1	179338327	T	0.08	-0.049	0.006	-0.052	0.011
rs10175975	2	59429807	T	0.19	0.025	0.004	0.016	0.008
rs10520176	2	77217310	T	0.49	0.038	0.004	0.039	0.006
rs1064213	2	198950240	A	0.48	0.044	0.004	0.065	0.006
rs113851554	2	66750564	T	0.06	-0.054	0.007	-0.067	0.014
rs11677484	2	191578172	T	0.26	0.023	0.004	0.018	0.007
rs11678584	2	32563426	A	0.86	-0.028	0.005	-0.041	0.009
rs11681299	2	88901732	T	0.28	0.024	0.004	0.035	0.007
rs12464387	2	75445544	A	0.46	-0.021	0.003	-0.024	0.006
rs12470914	2	50532840	A	0.10	0.053	0.006	0.069	0.010
rs13004345	2	174037347	T	0.65	-0.019	0.003	-0.025	0.006
rs13011556	2	4651923	C	0.76	-0.029	0.004	-0.025	0.007
rs13414393	2	54275162	T	0.54	-0.022	0.004	-0.021	0.006
rs17396357	2	48252311	T	0.38	0.021	0.004	0.032	0.006
rs184033703	2	206956138	A	0.06	0.058	0.008	0.054	0.013

rs2166559	2	149551658	T	0.86	-0.033	0.005	-0.025	0.009
rs2706762	2	70488470	T	0.15	-0.037	0.005	-0.045	0.008
rs28380327	2	144232491	A	0.63	0.040	0.004	0.052	0.006
rs34509802	2	101591710	A	0.18	0.040	0.005	0.051	0.008
rs359248	2	60477461	T	0.46	-0.028	0.003	-0.038	0.006
rs4666682	2	186203743	A	0.18	-0.025	0.004	-0.029	0.008
rs4672458	2	53736362	T	0.48	-0.022	0.003	-0.018	0.006
rs62124718	2	12822995	A	0.90	-0.045	0.006	-0.057	0.010
rs62182135	2	240267305	A	0.33	-0.024	0.003	-0.026	0.006
rs6433478	2	175241482	T	0.46	-0.025	0.004	-0.035	0.006
rs6544906	2	46863872	A	0.56	0.023	0.004	0.030	0.006
rs6727752	2	76361783	A	0.36	0.026	0.004	0.023	0.007
rs72796401	2	24180078	A	0.19	0.025	0.003	0.029	0.008
rs747003	2	161916409	T	0.61	0.020	0.003	0.017	0.006
rs75120545	2	44271496	T	0.03	0.086	0.010	0.085	0.018
rs76064513	2	125438641	T	0.13	0.034	0.006	0.042	0.009
rs77248969	2	136490731	A	0.11	-0.033	0.006	-0.034	0.009
rs80271258	2	239311505	T	0.08	-0.089	0.006	-0.096	0.011
rs812925	2	61680993	C	0.65	-0.031	0.004	-0.031	0.006
rs848552	2	36700580	C	0.48	-0.028	0.004	-0.033	0.006
rs111261826	3	7189617	A	0.68	-0.028	0.004	-0.031	0.006
rs111867612	3	157721819	A	0.10	-0.032	0.006	-0.033	0.010
rs112201801	3	82591379	T	0.93	-0.085	0.013	-0.085	0.013
rs114848860	3	36859494	A	0.97	-0.077	0.010	-0.095	0.021
rs12636669	3	50003323	T	0.08	0.057	0.006	0.071	0.011
rs13065394	3	132971327	T	0.29	-0.027	0.004	-0.024	0.007
rs1398346	3	110271943	T	0.87	0.026	0.005	0.032	0.009
rs1449403	3	85591467	A	0.12	0.042	0.006	0.055	0.009
rs1468945	3	185990392	A	0.79	-0.036	0.004	-0.035	0.007
rs149611468	3	8817423	T	0.99	0.143	0.018	0.174	0.032
rs1599374	3	160891727	A	0.52	0.031	0.004	0.037	0.006
rs17007397	3	70594975	C	0.58	0.023	0.004	0.026	0.006
rs1800828	3	113891549	C	0.75	0.026	0.004	0.016	0.007
rs2362775	3	24924421	T	0.53	-0.022	0.004	-0.008	0.006
rs301218	3	176096919	A	0.39	-0.024	0.004	-0.032	0.006
rs34967119	3	104778430	A	0.50	0.020	0.003	0.023	0.006
rs35346733	3	2521322	A	0.19	-0.032	0.005	-0.032	0.008
rs3850174	3	172364093	A	0.26	-0.035	0.004	-0.039	0.007
rs6440833	3	152646244	A	0.46	0.021	0.003	0.029	0.006
rs72950188	3	116103275	T	0.92	0.045	0.007	0.053	0.011

rs72966564	3	123149816	T	0.25	-0.023	0.004	-0.019	0.007
rs73050286	3	23224684	T	0.78	0.030	0.004	0.030	0.007
rs7429614	3	77205438	T	0.42	0.035	0.004	0.046	0.006
rs7626335	3	71575177	A	0.33	-0.029	0.004	-0.041	0.006
rs7649164	3	150788032	T	0.57	0.021	0.004	0.024	0.006
rs9817910	3	18246870	A	0.56	-0.022	0.003	-0.016	0.006
rs9836621	3	182096311	T	0.52	-0.028	0.004	-0.040	0.006
rs1502249	4	27495379	A	0.52	0.017	0.003	0.022	0.006
rs17455138	4	130903511	T	0.77	0.031	0.005	0.041	0.007
rs2850979	4	102094764	T	0.76	-0.023	0.004	-0.022	0.007
rs3796618	4	1349602	A	0.53	-0.023	0.004	-0.020	0.006
rs4241964	4	137053959	T	0.52	-0.029	0.003	-0.023	0.006
rs4690085	4	2697300	A	0.53	-0.019	0.003	-0.015	0.006
rs4698678	4	18260776	C	0.28	0.031	0.004	0.038	0.007
rs4860734	4	67096904	A	0.29	0.020	0.003	0.024	0.007
rs6816922	4	80206272	A	0.54	-0.020	0.004	-0.022	0.006
rs6838677	4	66520667	A	0.67	-0.021	0.004	-0.022	0.006
rs6846730	4	83279041	T	0.24	-0.032	0.004	-0.029	0.007
rs72729847	4	147296930	T	0.80	-0.030	0.005	-0.036	0.007
rs7700110	4	114439894	A	0.26	0.024	0.004	0.030	0.007
rs938836	4	139939653	A	0.47	-0.021	0.003	-0.027	0.006
rs9991917	4	132512118	A	0.19	0.045	0.008	0.045	0.008
rs9997394	4	163704083	A	0.29	-0.025	0.004	-0.031	0.007
rs10058356	5	35220404	T	0.70	-0.021	0.003	-0.018	0.006
rs12518401	5	173539588	A	0.38	-0.024	0.004	-0.034	0.006
rs13172141	5	122990902	A	0.57	0.022	0.004	0.028	0.006
rs1559253	5	106657015	A	0.36	0.022	0.004	0.036	0.006
rs2901796	5	163330708	A	0.40	0.025	0.004	0.026	0.006
rs42210	5	166408788	C	0.71	-0.029	0.005	-0.031	0.007
rs4269995	5	87701223	T	0.25	-0.034	0.004	-0.047	0.007
rs465670	5	176877624	T	0.54	0.024	0.004	0.023	0.006
rs67988891	5	152204741	C	0.68	-0.036	0.004	-0.031	0.006
rs7701529	5	63861475	A	0.24	-0.030	0.004	-0.029	0.007
rs7721608	5	76581258	T	0.46	0.020	0.003	0.030	0.006
rs7735794	5	175339984	A	0.22	0.034	0.006	NA	NA
rs77960	5	103964585	A	0.33	0.022	0.003	0.016	0.006
rs11154718	6	99592404	T	0.43	-0.023	0.004	-0.027	0.006
rs12195792	6	98705295	A	0.27	0.034	0.004	0.047	0.007
rs12206814	6	41517457	C	0.49	0.025	0.004	0.017	0.007
rs1811899	6	14878060	T	0.79	-0.030	0.005	-0.037	0.007

rs1931814	6	62589167	A	0.48	0.026	0.004	0.031	0.006
rs2050185	6	147936781	A	0.62	0.022	0.004	0.018	0.006
rs2396004	6	43355851	A	0.44	0.021	0.004	0.025	0.006
rs2653349	6	55142337	A	0.21	0.066	0.004	0.074	0.008
rs2881955	6	72479263	T	0.28	0.027	0.004	0.028	0.007
rs3857599	6	50938247	A	0.17	0.032	0.005	0.019	0.008
rs3923809	6	38440970	A	0.69	-0.022	0.004	-0.029	0.006
rs4535583	6	115699280	T	0.70	0.021	0.004	0.027	0.007
rs486416	6	31856070	A	0.65	-0.020	0.003	-0.020	0.006
rs60616179	6	110244765	A	0.94	0.051	0.008	0.031	0.013
rs766406	6	26319588	T	0.64	-0.024	0.004	-0.034	0.006
rs9347926	6	165195547	A	0.44	0.026	0.003	0.032	0.006
rs9348050	6	166263488	T	0.49	0.022	0.003	0.019	0.006
rs9381812	6	13183998	A	0.71	-0.050	0.004	-0.070	0.007
rs9394154	6	11574374	C	0.44	-0.022	0.003	-0.019	0.006
rs9465253	6	19102247	T	0.28	0.023	0.004	0.026	0.007
rs9479402	6	153135339	T	0.99	-0.219	0.017	-0.272	0.027
rs9496623	6	143751625	A	0.73	-0.024	0.004	-0.023	0.007
rs10237162	7	24085405	T	0.72	0.037	0.004	0.049	0.007
rs10254050	7	96468077	C	0.19	-0.058	0.005	-0.076	0.008
rs10951325	7	32265545	T	0.63	0.034	0.004	0.037	0.006
rs113161209	7	148564367	A	0.08	0.044	0.008	0.039	0.011
rs17302081	7	115673079	T	0.44	0.022	0.004	0.014	0.006
rs2072413	7	150647969	T	0.26	-0.021	0.004	-0.024	0.007
rs2944831	7	71779635	A	0.29	0.025	0.004	0.025	0.007
rs3807651	7	77823771	A	0.49	0.025	0.004	0.022	0.006
rs4027217	7	14093914	A	0.22	-0.026	0.005	-0.024	0.007
rs4236237	7	69936477	A	0.60	-0.024	0.004	-0.031	0.006
rs4729854	7	102383663	A	0.47	-0.049	0.004	-0.056	0.007
rs62465218	7	132294312	A	0.15	-0.027	0.005	-0.039	0.009
rs6958557	7	133585794	T	0.61	0.026	0.004	0.035	0.006
rs6967481	7	50642701	T	0.50	0.032	0.003	0.036	0.006
rs6968240	7	121942674	A	0.42	0.022	0.003	0.033	0.006
rs10109566	8	59800446	A	0.48	-0.022	0.004	-0.022	0.006
rs16939162	8	76653156	A	0.83	0.038	0.005	0.033	0.008
rs187028	8	73459513	A	0.31	-0.022	0.003	-0.028	0.006
rs1871729	8	136223702	A	0.68	-0.023	0.004	-0.026	0.006
rs2322605	8	27164449	A	0.47	-0.022	0.004	-0.025	0.006
rs2737245	8	116658583	T	0.27	0.034	0.004	0.035	0.007
rs2979139	8	8268313	A	0.50	-0.027	0.003	-0.030	0.006

rs3100052	8	101967139	A	0.39	0.025	0.004	0.021	0.006
rs34054660	8	65015659	A	0.57	0.025	0.004	0.033	0.006
rs35524253	8	4823608	A	0.35	0.034	0.004	0.047	0.006
rs62479736	8	3654320	T	0.29	0.024	0.004	0.026	0.007
rs6468316	8	35237788	T	0.47	-0.020	0.003	-0.015	0.006
rs6988733	8	91535686	T	0.35	0.023	0.004	0.025	0.006
rs6993892	8	33729200	T	0.62	-0.035	0.004	-0.037	0.006
rs7006885	8	93283578	A	0.29	0.030	0.004	0.050	0.007
rs71523448	8	31817493	C	0.08	-0.050	0.007	-0.063	0.012
rs7845620	8	53129069	A	0.83	-0.043	0.005	-0.058	0.008
rs10759208	9	109806199	T	0.61	-0.025	0.004	-0.026	0.006
rs10818834	9	126317324	T	0.73	0.030	0.004	0.026	0.007
rs10988239	9	131943440	T	0.51	-0.021	0.003	-0.016	0.006
rs11788633	9	116767656	C	0.65	0.020	0.003	0.029	0.006
rs12378543	9	83196097	T	0.38	-0.023	0.004	-0.022	0.006
rs12380242	9	139310187	T	0.51	-0.021	0.003	-0.016	0.006
rs2844016	9	24582747	T	0.29	0.027	0.004	0.031	0.007
rs295268	9	86429305	T	0.74	-0.031	0.005	-0.026	0.007
rs308521	9	37367094	T	0.60	0.028	0.003	0.031	0.006
rs3138490	9	92219000	A	0.52	0.024	0.004	0.023	0.006
rs4878734	9	38010085	A	0.51	0.022	0.004	0.016	0.006
rs555784	9	85318704	A	0.38	-0.025	0.004	-0.029	0.006
rs62553781	9	76679777	T	0.03	-0.069	0.009	-0.060	0.018
rs6477309	9	8450638	T	0.67	0.031	0.004	0.035	0.006
rs6560218	9	74245426	T	0.52	-0.022	0.004	-0.021	0.006
rs28458909	9	140257189	T	0.12	-0.070	0.006	-0.072	0.010
rs10762434	10	73044413	C	0.78	0.025	0.004	0.033	0.007
rs10830107	10	129304075	A	0.79	0.028	0.005	0.031	0.007
rs11200159	10	123553392	A	0.66	-0.023	0.004	-0.023	0.006
rs1163238	10	104943993	A	0.39	-0.024	0.004	-0.018	0.006
rs12249410	10	64301941	T	0.11	-0.034	0.006	-0.031	0.010
rs12771973	10	133749294	A	0.25	-0.022	0.004	-0.028	0.007
rs17712705	10	69623271	A	0.33	-0.025	0.004	-0.029	0.006
rs2298117	10	70346740	T	0.45	-0.023	0.004	-0.018	0.006
rs3808964	10	125426627	T	0.63	0.020	0.003	0.015	0.006
rs497338	10	804315	T	0.29	0.027	0.004	0.029	0.006
rs61875203	10	93888810	T	0.28	0.026	0.004	0.027	0.007
rs66617308	10	56699338	T	0.67	0.018	0.003	0.017	0.006
rs76518095	10	131149976	T	0.08	0.040	0.007	0.046	0.012
rs7900191	10	119145774	T	0.40	-0.019	0.003	-0.018	0.006

rs9416744	10	60567937	A	0.26	0.034	0.004	0.044	0.007
rs9664044	10	126710791	T	0.23	-0.027	0.004	-0.025	0.007
rs10742179	11	27650524	A	0.26	0.035	0.004	0.038	0.007
rs10832648	11	16618307	A	0.20	-0.031	0.004	-0.030	0.007
rs10838687	11	47312892	T	0.79	0.035	0.005	0.045	0.007
rs11032362	11	33759092	A	0.09	0.070	0.006	0.079	0.010
rs1278402	11	82972097	A	0.74	0.028	0.005	0.038	0.007
rs12808544	11	58373221	A	0.24	-0.035	0.004	-0.034	0.007
rs1508608	11	92893825	A	0.32	0.028	0.004	0.040	0.006
rs17577073	11	99152801	A	0.56	0.025	0.004	0.028	0.006
rs2514214	11	113395329	A	0.27	0.027	0.004	0.026	0.007
rs3867239	11	122093090	A	0.38	0.026	0.004	0.032	0.006
rs4121878	11	95120372	C	0.50	0.022	0.004	0.024	0.006
rs4923541	11	28479535	T	0.51	0.025	0.004	0.028	0.006
rs4936290	11	114009255	A	0.66	-0.023	0.003	-0.019	0.007
rs621421	11	30405914	T	0.63	-0.027	0.003	-0.029	0.006
rs662094	11	66342691	A	0.49	0.028	0.004	0.036	0.006
rs7111582	11	43893222	A	0.90	-0.039	0.005	-0.048	0.010
rs74357745	11	122811822	A	0.88	0.031	0.005	0.021	0.009
rs7943634	11	126734319	T	0.31	-0.024	0.004	-0.020	0.006
rs10877962	12	63520912	T	0.41	0.036	0.004	0.049	0.006
rs11611435	12	24089322	T	0.56	0.028	0.004	0.022	0.006
rs12298405	12	17015267	T	0.33	-0.023	0.004	-0.024	0.006
rs1799464	12	16286082	A	0.29	-0.020	0.004	-0.025	0.007
rs1843888	12	38737310	A	0.54	0.051	0.004	0.060	0.006
rs2433634	12	23060363	A	0.72	-0.027	0.004	-0.028	0.007
rs247929	12	46294908	C	0.51	0.031	0.004	0.036	0.006
rs3782860	12	361996	T	0.54	0.025	0.004	0.029	0.006
rs3955311	12	114343818	T	0.15	0.026	0.005	0.031	0.008
rs711098	12	77976559	A	0.40	0.022	0.004	0.033	0.006
rs7298532	12	112510404	T	0.72	0.027	0.004	0.025	0.007
rs7299922	12	54702519	A	0.64	0.024	0.004	0.023	0.006
rs7304278	12	106989915	A	0.28	-0.029	0.004	-0.030	0.007
rs7959983	12	90452978	T	0.60	-0.030	0.004	-0.028	0.006
rs7975791	12	49413486	T	0.04	0.051	0.009	0.054	0.016
rs80097534	12	121029604	T	0.10	-0.036	0.006	-0.024	0.011
rs1163628	13	112226420	A	0.86	-0.029	0.005	-0.033	0.008
rs1886205	13	94062095	A	0.76	0.029	0.004	0.034	0.007
rs2593487	13	69903058	A	0.34	-0.029	0.004	-0.034	0.006
rs3815983	13	109779906	T	0.36	-0.022	0.003	-0.025	0.006

rs45597035	13	73649152	A	0.65	-0.022	0.004	-0.023	0.006
rs495593	13	72919800	A	0.74	0.023	0.004	0.032	0.007
rs9558942	13	107700218	T	0.67	-0.019	0.003	-0.032	0.006
rs9571526	13	66590868	T	0.77	-0.027	0.005	-0.032	0.007
rs9573980	13	77590741	A	0.97	0.127	0.010	0.150	0.017
rs9597241	13	56281271	A	0.81	0.033	0.004	0.037	0.008
rs11845599	14	101016824	A	0.64	-0.027	0.004	-0.034	0.006
rs2878172	14	55373670	A	0.57	-0.021	0.004	-0.030	0.006
rs2978382	14	64769074	T	0.59	0.023	0.004	0.021	0.006
rs4550384	14	85350142	T	0.76	0.024	0.004	0.028	0.007
rs4903203	14	74660508	A	0.32	0.025	0.004	0.027	0.006
rs61990287	14	42069889	A	0.27	0.025	0.004	0.027	0.007
rs6573308	14	60806976	T	0.39	0.025	0.004	0.026	0.006
rs710284	14	98532540	T	0.58	0.022	0.004	0.025	0.006
rs7143933	14	62460219	T	0.26	0.025	0.004	0.034	0.007
rs962961	14	57281154	T	0.33	-0.022	0.003	-0.025	0.006
rs12442008	15	53725112	T	0.26	0.029	0.004	0.035	0.007
rs12442674	15	96907819	A	0.73	0.023	0.004	0.040	0.008
rs1873958	15	101147726	A	0.41	0.028	0.003	0.041	0.006
rs4775086	15	58969292	A	0.24	-0.027	0.005	-0.030	0.007
rs59986227	15	48009263	C	0.75	-0.031	0.004	-0.029	0.007
rs11641239	16	23124193	T	0.29	0.023	0.004	0.017	0.007
rs12445235	16	8195278	C	0.41	-0.021	0.004	-0.024	0.006
rs12927162	16	52684916	A	0.73	0.056	0.004	0.066	0.007
rs1421085	16	53800954	T	0.59	-0.042	0.003	-0.044	0.006
rs17604349	16	72210865	A	0.18	-0.037	0.004	-0.044	0.008
rs2304467	16	8988777	C	0.61	-0.024	0.004	-0.020	0.006
rs2550298	16	56367969	T	0.38	-0.040	0.004	-0.043	0.006
rs4785296	16	49467234	C	0.23	0.026	0.004	0.029	0.007
rs7203707	16	24518569	A	0.52	-0.020	0.003	-0.016	0.006
rs72773411	16	728514	A	0.15	0.029	0.005	0.035	0.009
rs72790386	16	68136932	T	0.03	0.060	0.011	0.071	0.017
rs8044054	16	60628436	T	0.39	0.031	0.004	0.031	0.006
rs1061032	17	8064083	T	0.09	0.064	0.006	0.065	0.010
rs11545787	17	17398278	A	0.25	-0.050	0.004	-0.070	0.007
rs12051	17	46103760	A	0.61	-0.026	0.004	-0.033	0.006
rs12600452	17	45054564	A	0.20	0.026	0.004	0.034	0.008
rs12950382	17	30603994	A	0.72	0.023	0.004	0.027	0.007
rs2011528	17	33980566	T	0.83	-0.033	0.005	-0.029	0.008
rs2916148	17	65482109	A	0.45	0.028	0.004	0.033	0.006

rs3760381	17	43047083	A	0.25	0.027	0.004	0.030	0.007
rs412000	17	56709058	C	0.56	-0.022	0.004	-0.025	0.006
rs4365329	17	31625887	A	0.54	-0.019	0.003	-0.025	0.006
rs55846845	17	50092201	A	0.52	-0.021	0.003	-0.021	0.006
rs58681483	17	57934654	A	0.92	0.035	0.006	0.055	0.010
rs72829706	17	54173733	A	0.96	0.056	0.009	0.052	0.015
rs72841368	17	61391114	A	0.81	-0.030	0.004	-0.030	0.008
rs8072058	17	55734198	A	0.78	-0.028	0.005	-0.031	0.007
rs1013987	18	22630836	T	0.40	-0.029	0.004	-0.022	0.006
rs1025601	18	73056278	T	0.39	-0.022	0.004	-0.016	0.006
rs11152350	18	60240352	A	0.47	-0.028	0.004	-0.029	0.006
rs12969848	18	38152835	T	0.53	0.036	0.004	0.036	0.006
rs1788784	18	21159630	A	0.34	-0.027	0.004	-0.043	0.006
rs2580160	18	1816036	A	0.56	0.028	0.004	0.024	0.006
rs34329963	18	64526233	T	0.11	-0.032	0.005	-0.039	0.009
rs4419127	18	31663654	A	0.66	0.044	0.004	0.060	0.006
rs4800998	18	53429655	A	0.18	0.039	0.005	0.057	0.008
rs62082402	18	5186566	T	0.19	0.050	0.005	0.060	0.011
rs9950528	18	35762461	A	0.65	-0.024	0.004	-0.025	0.006
rs9956387	18	44773382	A	0.50	-0.020	0.003	-0.018	0.006
rs9964420	18	56824041	A	0.30	-0.049	0.004	-0.064	0.007
rs10402849	19	2695661	T	0.20	0.026	0.004	0.020	0.008
rs11670534	19	47003906	T	0.16	-0.031	0.005	-0.026	0.008
rs36055559	19	5799433	A	0.17	-0.036	0.005	-0.045	0.010
rs56113850	19	41353107	T	0.42	-0.023	0.004	-0.031	0.006
rs58876439	19	42600984	A	0.07	0.047	0.007	0.053	0.012
rs7248205	19	10770305	T	0.60	0.027	0.004	0.032	0.006
rs73026775	19	31052954	A	0.12	-0.034	0.006	-0.041	0.010
rs9636202	19	18449238	A	0.27	-0.026	0.004	-0.023	0.007
rs1474754	20	20077178	A	0.26	-0.021	0.004	-0.029	0.007
rs1737893	20	31051699	T	0.38	-0.025	0.004	-0.028	0.006
rs2072727	20	43538733	T	0.43	0.028	0.003	0.033	0.006
rs57236847	20	44668401	C	0.60	0.027	0.004	0.030	0.006
rs6047481	20	21539564	A	0.67	0.025	0.004	0.025	0.006
rs6131805	20	16222093	T	0.40	0.026	0.004	0.026	0.006
rs6131942	20	17348608	A	0.42	-0.026	0.003	-0.033	0.006
rs139911	22	40704052	T	0.57	-0.034	0.004	-0.024	0.006
rs28459838	22	35846168	T	0.24	0.027	0.004	0.032	0.008
rs6007594	22	45728370	A	0.26	-0.025	0.004	-0.025	0.007
rs695459	22	28848278	T	0.39	-0.022	0.004	-0.019	0.006

Chr, chromosome; EA, effect allele; EAF, effect allele frequency; SE, standard error.

Supplementary Table 4. Results of multiple testing correction for combined associations by the false discovery rate (FDR) method

Cancer	Original P value	Critical Value	Benjamini-Hochberg Adjusted P value	Significant using an FDR of 0.05?
Digestive tract	0.007	0.007	0.050	Yes
Colorectum	0.009	0.014	0.031	Yes
Stomach	0.014	0.021	0.032	Yes
Biliary	0.108	0.029	0.189	No
Liver	0.525	0.036	0.735	No
Pancreas	0.849	0.043	0.991	No
Oesophagus	0.857	0.050	0.857	No

Supplementary Table 5. Associations between genetic liability to chronotype and cancers of digestive tract, stomach, and colorectum in the analysis where the exposure data were obtained from 23andMe

Cancer	Cochran's Q	$P_{intercept}$	Inverse variance weighted			Weighted median			MR-Egger			Contamination mixture		
			OR	95% CI	P	OR	95% CI	P	OR	95% CI	P	OR	95% CI	P
**UK Biobank**														
Digestive tract	318	0.561	0.96	0.91-1.01	0.085	0.94	0.87-1.02	0.161	0.99	0.88-1.11	0.844	0.96	0.91-1.01	0.145
Stomach	332	0.943	0.85	0.73-1.00	0.053	0.84	0.66-1.08	0.185	0.84	0.58-1.23	0.379	0.70	0.57-0.92	0.016
Colorectum	390	0.492	0.94	0.87-1.00	0.048	0.93	0.84-1.03	0.177	0.98	0.84-1.15	0.833	0.88	0.82-0.95	0.003
**FinnGen**														
Digestive tract	301	0.886	0.94	0.88-1.00	0.059	0.93	0.84-1.03	0.141	0.95	0.81-1.1	0.499	0.93	0.87-1.01	0.097
Stomach	292	0.301	0.89	0.75-1.06	0.204	1.00	0.75-1.33	0.992	1.09	0.72-1.65	0.692	1.07	0.81-1.40	0.559
Colorectum	307	0.429	0.94	0.86-1.02	0.133	0.94	0.82-1.08	0.402	1.01	0.83-1.23	0.944	0.87	0.79-0.96	0.009
**Meta-analysis**														
Digestive tract			0.95	0.91-0.99	0.012									
Stomach			0.87	0.77-0.98	0.022									
Colorectum			0.94	0.89-0.99	0.013									

CI indicates confidence interval; OR, odds ratio.
$P_{intercept}$ is the P for MR-Egger intercept test and a $P_{intercept}$ <0.05 indicates the horizonal pleiotropy.
